# Effects of sacral nerve magnetic stimulation on oxidative stress, inflammation and coagulation function in patients with neurogenic bladder after spinal cord injury

**DOI:** 10.5937/jomb0-61262

**Published:** 2026-04-15

**Authors:** Ruimin Wu, Jin Li

**Affiliations:** 1 Xi'an Honghui Hospital, Department of Thoracic Surgery and Urology, Xi'an, China; 2 Xi'an Honghui Hospital, Department of Otolaryngology-Head and Neck Plastic and Reconstructive Surgery, Xi'an, China

**Keywords:** spinal cord injury, neurogenic bladder, sacral nerve magnetic stimulation, oxidative stress, inflammatory factors, coagulation function, povreda kičmene moždine, neurogena bešika, magnetna stimulacija sakralnog nerva, oksidativni stres, inflamacija, hemostaza

## Abstract

**Background:**

This study investigated the effect of sacral nerve magnetic stimulation (SNM) on the neurogenic bladder (NB) following spinal cord injury (SCI). Pay close attention to the changes in serum oxidative stress factors, inflammatory factors and coagulation function.

**Methods:**

A total of 134 SCI-induced NB patients admitted between February 2021 and January 2025 were enrolled. All participants received information-motivation-behavioural skills (IMB) management, and patients were grouped according to their treatment regimen: a conventional group (n=71) receiving conventional treatment and an SNM group (n = 63) treated with SNM (20 Hz frequency, 1 Tesla intensity, 20-minute sessions, once a day, 5 times a week, for 4 consecutive weeks). Laboratory analyses included coagulation markers (D-dimer, fibrinogen), oxidative stress indicators (superoxide dismutase [SOD], glutathione peroxidase [GSH-Px], and malondialdehyde [MDA]), and inflammatory mediators (IL-1<span style="color: rgb(32, 33, 34); font-family: sans-serif; font-size: 16px; background-color: rgb(248, 249, 250);">β</span>, IL-6, TNF-<span style="color: rgb(32, 33, 34); font-family: sans-serif; font-size: 16px; background-color: rgb(248, 249, 250);">α</span>, and high-sensitivity C-reactive protein [hs-CRP]).

**Results:**

Coagulation function also improved, with lower D-dimer and fibrinogen levels in the SNM group (P&lt; 0.05). Regarding oxidative stress, the SNM group exhibited higher SOD and GSH-Px activity alongside reduced MDA levels (P&lt; 0.05). Furthermore, inflammatory markers (IL-6, TNF-<span style="color: rgb(32, 33, 34); font-family: sans-serif; font-size: 16px; background-color: rgb(248, 249, 250);">α</span>) were lower in the SNM group (P&lt; 0.05).

**Conclusions:**

SNM can alleviate the stress response and inflammatory response of patients with SCI-induced NB, relieve the hypercoagulable state of blood, and has certain clinical application value.

## Abstract (sr)

**Uvod:**

Cilj istraživanja je bio da se proceni efekat magnetne stimulacije sakralnog nerva (MSN) na neurogenu besiku (NB) kod bolesnika nakon povrede kičmene moždine (PKM), sa fokusom na promene u parametrima oksidativnog stresa, inflamacije i hemostaze.

**Metode:**

U studiju je uključeno 134 bolesnika sa NB izazvanom PKM, hospitalizovanih u periodu od februara 2021. do januara 2025. Svi ispitanici su bili obuhvaceni informaciono-motivaciono-bihevioralnim (IMB) programom, a prema terapijskom protokolu podeljeni su u dve grupe: konvencionalnu (n= 71), koja je primala standardnu terapiju, i MSN grupu (n = 63), tretiranu MSN (frekvencija 20 Hz, intenzitet 1 Tesla, trajanje 20 minuta, jednom dnevno, pet puta nedeljno, tokom četiri uzastopne nedelje). Laboratorijske analize su obuhvatale koagulacione pokazatelje (D-dimer, fibrinogen), markere oksidativnog stresa (superoksid-dismutaza [SOD], glutation-peroksidaza [GSH-Px], malondialdehid [MDA]) i inflamatorne citokine/medijatore (IL-1<span style="color: rgb(32, 33, 34); font-family: sans-serif; font-size: 16px; background-color: rgb(248, 249, 250);">β</span>, IL-6, TNF-<span style="color: rgb(32, 33, 34); font-family: sans-serif; font-size: 16px; background-color: rgb(248, 249, 250);">α</span>, visokoosetljivi C-reaktivni protein [hs-CRP]).

**Rezultati:**

U MSN grupi zabeleženo je značajno poboljšanje koagulacione funkcije, sa nižim vrednostima D-dimera i fibrinogena (P&lt; 0,05). Parametri oksidativnog stresa pokazali su visu aktivnost SOD i GSH-Px uz snižene koncentracije MDA (P&lt; 0,05). Inflamatorni markeri, posebno IL-6 i TNF-<span style="color: rgb(32, 33, 34); font-family: sans-serif; font-size: 16px; background-color: rgb(248, 249, 250);">α</span>, bili su značajno niži u odnosu na kontrolnu grupu (P &lt;0,05).

**Zaključak:**

Magnetna stimulacija sakralnog nerva može ublažiti oksidativni i inflamatorni odgovor, redukovati hiperkoagulabilno stanje i time doprineti poboljšanju kliničkog ishoda kod bolesnika sa neurogenom bešikom usled povrede kičmene moždine. Ova metoda pokazuje potencijalnu kliničku primenljivost u multidisciplinarnom lečenju ove populacije pacijenata.

## Introduction

Neurogenic bladder (NB) secondary to spinal cord injury (SCI) represents a prevalent clinical complication, with associated voiding dysfunction not only significantly impairing patients' quality of life but also predisposing them to severe secondary complications such as urinary tract infections and hydronephrosis, thereby posing multisystem health risks [1][2]. While conventional therapeutic approaches, such as intermittent catheterisation and anticholinergic medications, can provide partial symptomatic relief, their efficacy in promoting functional bladder recovery and addressing systemic pathophysiological imbalances remains limited [3]. In recent years, sacral nerve magnetic stimulation (SNM) has emerged as a promising non-invasive neuromodulation technique, demonstrating notable clinical outcomes by enhancing the excitability of the sacral micturition centre, thereby improving both bladder storage and voiding functions [4]. Nevertheless, the underlying mechanisms through which SNM mitigates systemic oxidative stress and coagulation dysfunction remain to be fully elucidated.

Research indicates that SCI induces systemic pathological changes extending far beyond localised neural damage. The oxidative stress imbalance exacerbates secondary neural tissue injury through the overproduction of reactive oxygen species (ROS) and activates platelet aggregation pathways, resulting in a hypercoagulable state [5]. Concurrently, inflammatory cytokine cascades further accelerate bladder wall fibrosis [6]. Current therapeutic research for NB primarily focuses on urodynamic improvements [7], with inadequate attention given to these systemic haematological abnormalities. Notably, SNM may modulate systemic redox homeostasis via the neuroendocrine-autonomic axis [8], while information-motivation-behavioural skills (IMB) interventions could indirectly ameliorate coagulation dysfunction associated with chronic stress by enhancing patient compliance and self-management capabilities [9]. However, whether the synergistic effects of these two interventions can provide dual »local-systemic« benefits through multitarget regulation remains unexplored in systematic studies.

In this study, we focused on analysing the effects of SNM on serum oxidative stress factors, inflammatory factors and coagulation function in patients with NB. Notably, we are the first to elucidate the intricate interplay between the oxidative stress, coagulation, and inflammation networks during NB treatment. The findings provide biomarker-based guidance for developing individualised rehabilitation strategies, demonstrating clinical application value and translational potential.

## Materials and methods

### Study design

This retrospective study analysed NB patients admitted to our hospital between February 2021 and January 2025. The sample size was determined using G*Power (version 3.1) with an effect size of 0.3, α = 0.05, β = 0.2, and a dropout rate of 10%, resulting in a total sample size requirement of 134 patients. Eligible participants were screened based on predefined inclusion and exclusion criteria, yielding a final cohort of 134 patients. All patients received IMB management [a characteristic nursing model of our hospital, including: (1) Education: bladder health education (weekly 30-minute sessions); (2) Motivation: goal-setting and self-efficacy training (monthly counseling); (3) Behavioural skills: bladder diary maintenance and clean intermittent catheterisation (CIC) training (daily) (implemented by certified nurses during their hospitalisation). Of these, 71 received conventional treatment (conventional group) and 63 underwent SNM therapy (SNM group).

### Inclusion and exclusion criteria

Inclusion criteria: Diagnosis of SCI according to the International Spinal Cord Injury Society (ISCoS) criteria [10], with injury level at T12-L2; Urodynamic confirmation of detrusor overactivity or detrusor-sphincter dyssynergia; Symptom duration 3 months; Age 18-75 years old; Clear consciousness [minimental status exam (MMSE) score 24 [11]] and intact cognitive function.

Exclusion criteria: Non-neurogenic bladder dysfunction; Severe coagulopathy [international normalised ratio (INR) >1.5 or activated partial thromboplastin time (APTT) >40s]; Use of antioxidants (vitamin C/E supplements within 3 months), anticoagulants, or immunosuppressants within the past 3 months; Contraindications to SNM therapy (e.g., cardiac pacemaker, spinal metallic implants); Hepatic or renal impairment; Pregnancy or lactation.

### Ethical considerations

The study protocol was approved by the Institutional Review Board of Xi'an Honghui Hospital (No. 2020-08-24-024-shen). Written informed consent was obtained from all participants, and the research was conducted in accordance with the ethical principles outlined in the Declaration of Helsinki. Due to the nature of SNM intervention, blinding of participants and therapists was not feasible. Outcome assessors were blinded to group allocation. There were no dropouts, and the last observation carried forward was used for the intention-to-treat analysis.

### Methods

After admission, all patients received conventional treatment as prescribed by doctors. The SNM group added SNM (20 Hz frequency, 1 Tesla intensity, 20-minute sessions, once a day, 5 times a week, for 4 consecutive weeks) to the conventional treatment.

### Laboratory tests

Fasting venous blood samples were collected before and after treatment and divided into three aliquots. The first aliquot was analysed using a fully automated coagulation analyser (C2000-A, Mindray) to assess prothrombin time (PT), activated partial thromboplastin time (APTT), thrombin time (TT), D-dimer (D-D), and fibrinogen (FIB): The plasma was mixed with the corresponding coagulation indicators, and the instrument automatically recorded the reaction time and output the results. Quality control: commercial quality control plasma (matching quality control, target value range: PT 11-13s, APTT 28-34s, D-D 0.3-0.8 μg/mL) was used.

The second aliquot was processed using a fully automated haematology analyser (BS-20s, Mindray) to determine high-sensitivity C-reactive protein (hs-CRP) levels. The plasma was mixed with reagent 1 (buffer), and then reagent 2 (anti-CRP latex particle antibody) was added. The mixture was incubated at 37°C for 5 minutes. The latex particles were combined with CRP to form agglutination, and the absorbance change at 340 nm was detected by nephelometry and quantified against the standard curve (detection range: 0.1-100 mg/L). Quality control: the quality control samples were tested in the same batch as the patient samples, and the CV (coefficient of variation) was required to be 3% (within batch) and 5% (between batch).

The third aliquot was centrifuged to isolate serum. Enzyme-linked immunosorbent assay (ELISA) kits were employed to quantify superoxide dismutase (SOD) (kit: Beijing Boosen Biotechnology Co., LTD., BSKH62259), glutathione peroxidase (GSH-Px) (kit: Guangzhou Orida Biotechnology Co., LTD., ARD 10797), malondialdehyde (MDA) (kit: Beijing Boosen Biotechnology Co., LTD., EF007363), interleukin-1β (IL-1β) (kit: Guangzhou Orida Biotechnology Co., LTD., ARD10092), interleukin-6 (IL-6) (kit: Beijing Boaosen Biotechnology Co., LTD., bsk11007), and tumor necrosis factor-α (TNF-α) (kit: Beijing Boaosen Biotechnology Co., LTD., bsk11014). Microplates were pre-coated with specific capture antibodies, followed by the addition of the sample and detection antibodies. Colour development was performed, and absorbance was measured at 450 nm using a microplate reader. A four-parameter Logistic curve was fitted using the log value of the concentration of the standard as the X-axis and the log-log value of the OD value as the Y-axis, and the concentration was calculated by substituting the OD value of the sample to be tested. The washing machine was regularly calibrated (pressure, soaking time) to ensure consistent washing (residual rate 1%).

### Statistical analysis

Data analysis was conducted using SPSS 24.0. Categorical variables were expressed as frequencies and percentages [n (%)] and analysed with the chi-square test. For normally distributed continuous variables, results were expressed as (χ̄±s) and analysed using independent/paired t-tests. Non-normally distributed data were presented as medians (interquartile ranges, IQR) and compared using the Mann-Whitney U or Wilcoxon rank-sum tests. Post-hoc pairwise comparisons were adjusted using the Bonferroni method (a adjusted to 0.017 for three comparisons). Statistical significance was defined as *P*<0.05.

## Results

### Comparability analysis

Baseline characteristics, including age, gender, and disease duration, were compared between the two study groups. No statistically significant differences were observed (*P*>0.05), confirming the comparability of the groups (Table 1).

**Table 1:** Clinical data.

	Conventional group	SNM group	Statistical<br>values	*P* values
	n=71	n = 63		
Age (years)	57.35±3.90	58.54±4.95	t=1.551	0.123
Male/female	61 (85.92)/10 (14.08)	58 (92.06)/5 (7.94)	χ^2^ = 1.269	0.260
BMI (kg/m^2^)	25.10±2.11	25.15±2.48	t=0.120	0.905
Duration of disease (months)	4.94±1.64	5.16±1.57	t=0.774	0.440
ASIA classification			χ^2^=0.715	0.699
B/C/D	26 (36.62)/32 (45.07)/13	19 (30.16)/30 (47.62)/14		
Smoking	45 (63.38)	42 (66.67)	χ^2^=0.158	0.691
Alcohol consumption	32 (45.07)	27 (42.86)	χ^2^=0.066	0.797
Bladder history			χ^2^=0.012	0.914
yes/no	6 (8.45)/65 (91.55)	5 (7.94)/58 (92.06)		
Initial bladder sensation (mL)	132.08±21.45	137.47±25.72	1.321	0.189
Maximum bladder capacity (mL)	290.92±41.20	293.61±41.73	0.376	0.708
Maximum bladder pressure (mL)	18.64±6.83	18.73±4.66	0.086	0.932
Residual urine volume (mL)	182.66±40.39	184.05±21.67	0.244	0.808

### Comparison of coagulation function before and after treatment

Baseline coagulation parameters were comparable between the two groups (*P*>0.05). Post-treatment, both groups showed reductions in D-D and FIB levels, with the SNM group exhibiting even lower values than the conventional group (*P*<0.05). Additionally, PT, APTT, and TT decreased in both groups compared to baseline (*P*<0.05), although no significant intergroup differences were detected (*P*>0.05; Table 2).

**Table 2:** Coagulation function. Note: * indicates P < 0.05 for within-group comparisons (vs. baseline data).

		Conventional group	SNM group	t values	*P* values
		n=71	n = 63		
PT (s)	baseline	13.87±2.95	13.49±2.26	0.848	0.398
	After	9.22±2.10^*^	8.98±2.06^*^	0.662	0.509
APTT (s)	baseline	38.41±3.96	38.60±5.69	0.219	0.827
	After	35.05±3.34^*^	34.29±4.84^*^	1.060	0.291
TT (s)	baseline	16.70±2.95	16.49±.79	0.423	0.673
	After	13.43±2.19^*^	13.16±2.24^*^	0.715	0.476
D-D (μg/mL)	baseline	0.84±0.23	0.81±0.24	0.877	0.382
	After	0.74±0.18^*^	0.63±0.17^*^	3.798	<0.001
FIB (g/L)	baseline	3.52±0.54	3.61±0.59	0.936	0.351
	After	3.08±0.40^*^	2.69±0.45^*^	5.309	<0.001

### Comparison of serum oxidative stress markers before and after treatment

Both groups demonstrated improvements in oxidative stress parameters following treatment. Specifically, SOD and GSP-Px levels increased from baseline, while MDA levels decreased (*P*<0.05). Notably, the intergroup analysis revealed that the SNM group achieved superior outcomes compared to the conventional group, with higher post-treatment SOD and GSP-Px levels (*P*<0.05) and lower MDA levels (*P*<0.05, Table 3).

**Table 3:** Oxidative stress response. Note: * indicates P < 0.05 for within-group comparisons (vs. baseline data).

		Conventional group	SNM group	t values	*P* values
		n=71	n = 63		
SOD (U/mL)	baseline	126.06±2037	127.70±17.46	0.499	0.619
	After	134.94±18.75^*^	144.40±17.67^*^	2.993	0.003
GSH-Px (U/L)	baseline	5742.11±251.26	5799.316.98	1.176	0.242
	After	6150.14±342.50^*^	6460.23±298.17^*^	5.735	<0.001
MDA (μmol/L)	baseline	7.02±1.39	7.28±1.64	1.023	0.308
	After	6.40±1.36^*^	5.66±1.24^*^	3.261	0.001

### Comparison of serum inflammatory markers before and after treatment

Finally, the comparison of inflammatory markers between the two groups revealed no significant differences at baseline (*P*>0.05). Post-treatment evaluation revealed a reduction in inflammatory markers in both groups (*P*<0.05), indicating effective suppression of inflammatory responses. While IL-1β and hs-CRP levels did not differ significantly between groups (*P*>0.05), the SNM group demonstrated lower concentrations of IL-6 and TNF-α compared to the conventional group (*P*<0.05, Table 4).

**Table 4:** Inflammatory response. Note: * indicates P < 0.05 for within-group comparisons (vs. baseline data).

		Conventional group	SNM group	t values	P values
		n=71	n = 63		
IL-1β (pg/mL)	baseline	11.33±2.32	12.04±2.26	1.804	0.074
	After	9.42±1.66^*^	9.05±1.85^*^	1.216	0.226
IL-6 (pg/mL)	baseline	10.37±1.55	9.99±1.83	1.299	0.196
	After	9.28±1.72*	8.51±1.50^*^	2.734	0.007
TNF-α (pg/mL)	baseline	18.69±3.28	18.40±3.31	0.516	0.607
	After	13.80±3.00^*^	12.61±2.92^*^	2.325	0.022
hs-CRP (mg/L)	baseline	7.54±2.49	7.03±2.23	1.225	0.223
	After	5.75±2.16^*^	5.54±2.28^*^	0.542	0.589

## Discussion

This study provides the first comprehensive assessment of the therapeutic efficacy of SNM in systemic pathophysiological parameters in patients with NB secondary to SCI. Our findings indicate that the SNM group achieved better outcomes than the conventional treatment group in terms of symptom relief, coagulation function, oxidative stress response, and inflammatory markers. These results offer valuable implications for optimising NB management strategies.

First, we observed that SNM markedly increased the activity of SOD and GSH-Px while reducing MDA levels in NB patients. This demonstrates that SNM effectively counteracts oxidative stress imbalance following SCI. The potential mechanisms underlying this effect may include: (1) Activation of the Nrf2/ARE pathway: Emerging evidence suggests the nuclear factor erythroid 2-related factor 2 (Nrf2)/antioxidant response elements (ARE) pathway may mediate antioxidant effects in SCI models [4], though in vivo validation in NB patients is pending. (2) Inhibition of NADPH oxidase: Magnetic stimulation may attenuate NADPH oxidase complex activity, reducing ROS generation and subsequent lipid peroxidation products such as MDA [12]. Regarding coagulation function, the SNM group exhibited a greater decrease in D-D and FIB levels after treatment, confirming SNM's specific role in improving hypercoagulability. This effect may be mediated through SNM's suppression of excessive sacral sympathetic nerve activity, leading to reduced production of platelet-activating factor (PAF) and thrombin, ultimately lowering blood hypercoagulability [13]. Additionally, an animal study by Xu X et al. [14] demonstrated that magnetic stimulation suppresses tissue factor (TF) expression in vascular endothelial cells, effectively inhibiting the activation of the extrinsic coagulation pathway. This suggests that SNM may influence coagulation function by modulating vascular endothelial activity, though further investigation is needed to confirm this mechanism. In terms of inflammatory markers, the SNM group exhibited reduced levels of IL-6 and TNF-α, indicating that SNM may attenuate systemic inflammation through multiple pathways. One possible mechanism involves the vagus nerve-cholinergic anti-inflammatory pathway, where SNM suppresses nuclear factor kappa B (NF-B) translocation, thereby decreasing the release of pro-inflammatory cytokines (e.g., IL-6, TNF-α) [15]. Furthermore, studies suggest that magnetic stimulation promotes macrophage polarisation toward the M2 anti-inflammatory phenotype, which could alleviate bladder wall fibrosis [16] - further supporting SNM's anti-inflammatory role. Although IL-1β and hs-CRP levels improved in both groups, no between-group differences were observed. This may reflect ceiling effects of these acute-phase reactants or insufficient SNM-mediated modulation of systemic inflammation pathways. Evidence has indicated that SNM may specifically regulate the sympathetic-parasympathetic balance [17], potentially optimising bladder storage-voiding cycles and reducing involuntary detrusor contractions. However, as this study did not assess neurotransmitter levels, this hypothesis remains to be validated. Notably, the potential contribution of IMB management should also be acknowledged. By integrating its three core components - information, motivation, and behavioral skills - the IMB intervention enhanced patients' compliance with complex treatment protocols (e.g., the IMB model may indirectly mitigate chronic inflammatory stimulation by improving patients' treatment adherence and confidence [18], thereby providing behavioral medicine support for SNM's anti-inflammatory effects), highlighting its unique value as an adjunct to behavioral therapy.

In future clinical practice, we propose that SNM should be considered a first-line rehabilitation strategy for NB secondary to SCI. This approach should be prioritised for patients presenting with systemic pathological disturbances, such as hypercoagulability or oxidative stress. To optimise patient outcomes, a multidisciplinary team comprising urologists, rehabilitation specialists, and psychologists should collaborate to develop individualised treatment plans. It is essential to note that the current findings are derived from a single-centre study with a limited sample size, which may increase the risk of spurious outcomes. Additionally, although this study demonstrated short-term efficacy over 4 weeks, longer follow-up is necessary to assess the sustained effects on thromboembolic complications and bladder remodelling. Future investigations should incorporate mechanistic studies to elucidate the underlying pathways of SNM in the management of NB. Further validation through animal experiments is also warranted to clarify the mechanisms of action of SNM in this patient population.

## Conclusion

SNM is recommended as an adjunctive therapy for patients with SCI-associated NB exhibiting hypercoagulability, oxidative stress, or refractory detrusor overactivity, ideally initiated within 6 months post-injury. This multi-target therapeutic approach offers novel insights into the interactive regulation of the oxidative stress-coagulation-inflammation network, underscoring its considerable potential for clinical translation.

## Dodatak

### Data availability statement

The original data presented in the study are included in the article. For further inquiries, please get in touch with the corresponding authors.

### Acknowledgements

None.

### Conflict of interest statement

All the authors declare that they have no conflict of interest in this work.

## References

[1] Jiang Y, Li X, Guo S, Wei Z, Xu S, Qin H, Xu J (2024). Transcutaneous Electrical Stimulation for Neurogenic Bladder After Spinal Cord Injury: A Systematic Review and Meta-Analysis. Neuromodulation.

[2] Panicker J N (2020). Neurogenic Bladder: Epidemiology, Diagnosis, and Management. Semin Neurol.

[3] Bapir R, Bhatti K H, Eliwa A, García-Perdomo H A, Gherabi N, Hennessey D, Magri V, Mourmouris P, Ouattara A, Perletti G, Philipraj J, Stamatiou K, Trinchieri A (2022). Efficacy of overactive neurogenic bladder treatment: A systematic review of randomized controlled trials. Arch Ital Urol Androl.

[4] Wu S, Sun X, Liu X, Li J, Yang X, Bao Y, Yu H (2022). Clinical Observations of Percutaneous Tibial Nerve Stimulation Combined with Sacral Nerve Root Magnetic Stimulation for the Treatment of Male Chronic Pelvic Pain and Chronic Prostatitis. Arch Esp Urol.

[5] Pedroza-García K A, Careaga-Cárdenas G, Díaz-Galindo C, Quintanar J, Hernández-Jasso I, Ramírez-Orozco R E (2023). Bioactive role of vitamins as a key modulator of oxidative stress, cellular damage and comorbidities associated with spinal cord injury (SCI). Nutr Neurosci.

[6] Wang R, Wang M, Chen J, Sun M, Zhao P, Zheng X, Li D, Long C, Shen L, Wei G, Wu S (2025). Targeting STING alleviates pyroptosis of bladder epithelial cells and ameliorates bladder fibrosis in neurogenic bladder. Biochem Pharmacol.

[7] Manaila A I, Roman N A, Baseanu I C C, Minzatanu D, Tuchel V I, Basalic E B, Miclaus R S (2024). The Efficiency of Rehabilitation Therapy in Patients Diagnosed with Neurogenic Bladder: A Systematic Review. Medicina (Kaunas).

[8] Zhao Y, Wang D, Zou L, Mao L, Yu Y, Zhang T, Bai B, Chen Z (2022). Comparison of the efficacy and safety of sacral root magnetic stimulation with transcutaneous posterior tibial nerve stimulation in the treatment of neurogenic detrusor overactivity: An exploratory randomized controlled trial. Translational Andrology and Urology.

[9] Littner L, Thomas E, Doyle J, Hendrickson J, Adkins S, Mooney L, Tarango C (2023). Improving bleeding disorder treatment log adherence: An application of the information-motivation-behavioral skills model. Haemophilia.

[10] Snider B A, Eren F, Reeves R K, Rupp R, Kirshblum S C (2023). The International Standards for Neurological Classification of Spinal Cord Injury: Classification Accuracy and Challenges. Top Spinal Cord Inj Rehabil.

[11] Jia X, Wang Z, Huang F, Su C, Du W, Jiang H, Wang H, Wang J, Wang F, Su W, Xiao H, Wang Y, Zhang B (2021). A comparison of the Mini-Mental State Examination (MMSE) with the Montreal Cognitive Assessment (MoCA) for mild cognitive impairment screening in Chinese middle-aged and older population: A cross-sectional study. BMC Psychiatry.

[12] Li J, Tang C, Yang L, Song C, Wang Y (2025). The effect of sacral nerve root magnetic stimulation on bladder urodynamics and M3 receptor expression in rats with neurogenic bladder. IBRO Neuroscience Reports.

[13] Kale A, Baydili K N S, Keles E, Gundogdu E, Usta T, Oral E (2022). Comparison of Isolated Sciatic Nerve and Sacral Nerve Root Endometriosis: A Review of the Literature. J Minim Invasive Gynecol.

[14] Xu X, Li F, Liu C, Wang Y, Yang Z, Xie G, Zhang T (2024). Low-frequency repetitive transcranial magnetic stimulation alleviates abnormal behavior in valproic acid rat model of autism through rescuing synaptic plasticity and inhibiting neuroinflammation. Pharmacol Biochem Behav.

[15] Zuo C, Cao H, Feng F, Li G, Huang Y, Zhu L, Gu Z, Yang Y, Chen J, Jiang Y, Wang F (2022). Repetitive transcranial magnetic stimulation exerts anti-inflammatory effects via modulating glial activation in mice with chronic unpredictable mild stress-induced depression. Int Immunopharmacol.

[16] Lu Y, Chen K, Zhao W, Hua Y, Bao S, Zhang J, Wu T, Ge G, Yu Y, Sun J, Zhang F (2023). Magnetic vagus nerve stimulation alleviates myocardial ischemia-reperfusion injury by the inhibition of pyroptosis through the M2AChR/OGDHL/ROS axis in rats. J Nanobiotechnology.

[17] Zhang X, Zhu L, Li Y, Yu H, Wang T, Chu X (2025). Therapeutic potential and mechanisms of repetitive transcranial magnetic stimulation in Alzheimer's disease: A literature review. Eur J Med Res.

[18] Ma L, Yu Y, Zhao B, Yu Y, Li Y (2024). Effect of information-motivation-behavioral skills model based perioperative nursing on pain in patients with gallstones. World J Gastrointest Surg.

